# Cytomegalovirus infection may be oncoprotective against neoplasms of B-lymphocyte lineage: single-institution experience and survey of global evidence

**DOI:** 10.1186/s12985-022-01884-1

**Published:** 2022-09-29

**Authors:** Marko Janković, Aleksandra Knežević, Milena Todorović, Irena Đunić, Biljana Mihaljević, Ivan Soldatović, Jelena Protić, Nevenka Miković, Vera Stoiljković, Tanja Jovanović

**Affiliations:** 1 Institute of Microbiology and Immunology, Department of Virology, Faculty of Medicine, University of Belgrade, dr Subotića 1, Belgrade, 11000 Republic of Serbia; 2 Clinic for Hematology, Faculty of Medicine, University Clinical Centre of Serbia, University of Belgrade, dr Koste Todorovića 2, Belgrade, 11000 Republic of Serbia; 3 Institute of Medical Statistics and Informatics, Faculty of Medicine, University of Belgrade, dr Subotića 15, Belgrade, 11000 Republic of Serbia; 4 Institute of Virology, Vaccines, and Sera “Torlak”,, Vojvode Stepe 458, Belgrade, 11152 Republic of Serbia

**Keywords:** Cytomegalovirus, B-cell malignancies, Global, Seroprevalence, Oncoprotection

## Abstract

**Background:**

Although cytomegalovirus (CMV) is not considered tumorigenic, there is evidence for its oncomodulatory effects and association with hematological neoplasms. Conversely, a number of experimental and clinical studies suggest its putative anti-tumour effect. We investigated the potential connection between chronic CMV infection in patients with B-lymphocyte (B-cell) malignancies in a retrospective single-center study and extracted relevant data on CMV prevalences and the incidences of B-cell cancers the world over.

**Methods:**

In the clinical single-center study, prevalence of chronic CMV infection was compared between patients with B-cell leukemia/lymphoma and the healthy controls. Also, global data on CMV seroprevalences and the corresponding country-specific incidences of B- lineage neoplasms worldwide were investigated for potential correlations.

**Results:**

Significantly higher CMV seropositivity was observed in control subjects than in patients with B-cell malignancies (*p* = 0.035). Moreover, an unexpected seroepidemiological evidence of highly significant inverse relationship between country-specific CMV prevalence and the annual incidence of B-cell neoplasms was noted across the populations worldwide (ρ = −0.625, *p* < 0.001).

**Conclusions:**

We try to draw attention to an unreported interplay between CMV infection and B-cell lymphomagenesis in adults. A large-scale survey across > 70 countries disclosed a link between CMV and B-cell neoplasms. Our evidence hints at an antagonistic effect of chronic CMV infection against B-lymphoproliferation.

## Background

Although a benign infectious agent in the healthy, the human cytomegalovirus (CMV) is a notorious driver of morbidity and mortality in hematological patients with failed immunocompetence [1]. Cytomegalovirus infection is the most significant viral complication of allogeneic hematopoietic cell transplantation (HCT) [2, 3]. The virus is highly pervasive, with a widely varying seroprevalence due to different demographic factors including socioeconomic status (SES) of populaces and communities [4–6].

While not regarded as a *bona fide* tumorigenic virus, CMV boasts an array of features that imply its oncogenic potential. The genome of CMV carries two anti-apoptotic genes, upregulates p53 [7] and augments anaplasia in cancer cells and/or tumor-associated cells [8–10]. Also, CMV may contribute to cancer formation via a “hit-and-run” mechanism, as well [11–14]. Aditionally, recent studies have identified congenital CMV infection as a risk for developing childhood hematologic malignancy [15, 16].

In contrast, clinical evidence that favors an anti-limphoproliferative effect of CMV, recently came from de Carvalho Batista Éboli et al*.* (2022). They verified liver pretransplant positivity for CMV as a protective factor for posttransplant lymphoproliferative disorder (PTLD) in pediatric patients [17]. A possible virus-*vs*-leukemia phenomenon has also been described [18], along with inhibition of migration of tumor cells [19, 20]. Several experiments done with murine CMV documented apoptosis in tumor cells [21, 22]. In humans, patients experiencing CMV reactivation early after allogeneic HCT for acute leukemia and non-Hodgkin lymphomas (NHL) have reduced relapse rates [23–27].

Research on CMV infection, reactivation, and multiorgan sequelae preferentially focuses on T-lymphocyte (T-cell) immune response. Recent studies on humanized animal models make the case in favor of importance of anti-CMV antibodies as being produced by host B-cells [28–32].

We asked if CMV seroststus may relate to a possible oncomodulatory role played by chronic CMV infection in individuals afflicted by lymphoid neoplasias derived from a single histologic lineage. The current work provides evidence that chronic CMV infection protects against malignant diseases of B-lymphocyte origin.

## Methods

### Patient and control cohorts

Our retrospective study cohort (*N* = 83; M/F = 43/40) was monocentric and comprised patients treated at the Clinic of Hematology, University Clinical Center, Belgrade, Republic of Serbia. The median age was 49.45 years (M = 52.3, range 20–73; F = 48.1, range 21–73). Information on demographic markers, underlying B-cell disorders, and chemoradiation regimens administered was abstracted from patients' medical records. Principal patient characteristics, diagnoses and chemotherapy regimens are presented in Tables 1 and 2. Close relatedness of malignant diseases with B-lymphocyte ontogeny was considered to have a virological authority over the clinical diversity of B-cell neoplasms.

**Table 1:** Principal demographics, clinical characteristics, and CMV serology of the patient group

	IgG positive	IgG negative	*p*-value
Patients (N = 83)	75 (90.4%)	8 (9.6%)	N/A
Age (median, years)	50.05	43.75	0.070^a^
*Age categories (years)*			
20–39	18 (85.7%)	3 (14.3%)	0.054^b^
40–59	34 (89.5%)	4 (10.5%)	
> 59	23 (95.8%)	1 (4.2%)	
*Gender*			
Male	37 (86%)	6 (14%)	0.266^c^
Female	38 (95%)	2 (5%)	
Diagnose; ICD-O-3 code*			
Non-Hodgkin lymphoma; 9591/3	33 (94.3%)	2 (5.7%)	0.339^c^
Hodgkin’s disease; 9650/3	18 (81.8%)	4 (18.2%)	
B-chronic lymphocytic leukemia; 9823/3	9 (90%)	1 (10%)	
Waldenström macroglobulinemia; 9761/3	2 (100%)	0	
B-cell lymphoma, NOS; 9690/3	2 (100%)	0	
Plasma cell myeloma; 9732/3	2 (66.7%)	1 (33.7%)	
Hairy cell leukemia; 9940/3	2 (100%)	0	
B-cell acute lymphoblastic leukemia; 9811/3	7 (100%)	0	
Antiviral therapy (Acyclovir)**			
Yes	17 (81%)	4 (19%)	0.438^c^
No	36 (89.5%)	4 (10.5)	
Chemotherapy^†^			
Yes	38 (92.7%)	3 (7.3%)	0.706^c^
No	25 (83.3%)	5 (16.7%)	

*Diagnostic recognition and technical signification of lymphoid B-cell dyscrasias listed were morphologically code-specified and comply with criteria of the Classification of Diseases for Oncology (World Health Organization) and the 2016 revision of lymphoid neoplasms [35, 69]

**Data missing in 22 patients

^†^Data missing in 12 patients

^a^Mann–Whitney *U* test

^b^Mantel–Haenszel chi square test for trend

^c^Fisher's Exact Test

**Table 2:** Details on chemotherapy

Regimens*	Patients (*N* = 83)
ABVD	4
BEAM	1
CHOP	4
COP	1
DHAP	10
R-DHAP	1
Endoxan	1
ESHAP	1
PAD	1
R-EPOCH	1
R-CHOP	8
HyperCVAD	8
Untreated	42

*ABVD—Doxorubicin hydrochloride (Adriamycin), Bleomycin sulfate, Vinblastine sulfate, and Dacarbazine; *BEAM*—Carmustine (BiCNU), Etoposide, Cytarabine (Ara-C, cytosine arabinoside), Melphalan; *R-CHOP*—Rituximab, Cyclophosphamide, Doxorubicin hydrochloride, Vincristine (Oncovin), Prednisolone; *CTD*—Cyclophosphamide (Endoxan), Thalidomide, Dexamethasone; *DHAP*—Dexamethasone, High-dose Ara-C, Platinol (cisplatin); *ESHAP*—Etoposide, Methylprednisolone, High-dose Ara-C, Cisplatin; *PAD*—Bortezomib, Doxorubicin, Dexamethasone; *R-CD*—Rituximab, Cyclophosphamide, Dexamethasone; *P-CVP*—Rituximab, Cyclophosphamide, Vincristine, Prednisolone; *R-EPOCH*—Rituximab, Etoposide, Prednisone, Vincristine, Cyclophosphamide, Doxorubicin; *HyperCVAD*—Cytarbine, Vincristine, Cyclophosphamide, Doxorubicine, Dexamethasone

The control group (N = 259; M/F = 73/186) consisted of population-based pauci-symptomatic noninstitutionalized civilians (mean age: 41.79 years, range: 20–86). None among the controls has had a record of malignant disease. Study cohorts differed substantially by age and gender (*p* < 0.001) requiring statistical matching (Table 3).

**Table 3:** Statistical information on patient and control groups prior and after matching for age and gender

Cohort characteristics	Unadjusted			Matched cases		
	Patients	Controls	*p*-value	Patients	Controls	*p*-value
Subjects (*N*)	83	259	N/A	83	77	N/A
Mean age (yr.) range	49.45 20–73	41.79 20–86	< 0.001*	49.45 20–73	48.05 22–86	0.551*
*Gender (N, %)*						
Male	43 (51.8%)	73 (28.2%)	** < 0.001**^†^	43 (51.8%)	29 (37.7%)	0.072^†^
Female	40 (48.2%)	186 (71.8%)		40 (48.2%)	48 (62.3%)	
*CMV IgG (N, %)*						
Yes	75 (90.4%)	214 (82.6%)	0.09^†^	75 (90.4%)	76 (98.7%)	**0.035**^†^
No	8 (9.6%)	45 (17.4%)		8 (9.6%)	1 (1.3%)	

*Mann–Whitney *U* Test

^†^Fisher’s Exact Test

### Sampling and data collection

Whole blood was a clinical source of samples collected between February and November 2017 by venipuncture using standardized clot-activator vacutainers. After clotting and centrifugation the serum fraction was screened for anti-CMV IgG and IgM antibodies at the Virology Laboratory of the Institute of Microbiology and Immunology, Faculty of Medicine, University of Belgrade. Antibody classes were determined by means of commercial anti-CMV ELISA IgG and IgM kits (EUROIMMUN AG, Lübeck, Germany), with antibody detection performed spectrophotometrically on an ELISA Reader 270 (bioMérieux, Marcy-l’Étoile, France).

Peripheral blood samples from control cohort members were profiled for the presence of anti-CMV antibodies at the Institute of Virology, Vaccines, and Sera “Torlak”, Serbian National Reference Laboratory for Viruses. Commercial kits (Enzygnost, Marburg, Germany) and a Multiskan Ex ELISA Reader (Thermo Electron Corporation, Waltham, MA, USA) were used to detect IgG and IgM classes.

Prevalence of IgG seropositivity is a hallmark of past infections in a population [6]. Cytomegalovirus positivity was based on detection of either CMV specific IgG or both IgG and IgM in the serum indicating contact with the pathogen. All persons presenting with an antibody profile consistent with primoinfection were excluded from the study, as it was posited that there is not enough time for a B-cell malignancy to develop within a first-contact millieu.

Diseases herein studied belonged strictly to B-cell immunolymphoproliferative disorders. The diagnoses were established and morphologically code-specified according to the International Classification of the Diseases for Oncology by World Health Organization, ICD-0-3 [33] and the 2016 revision of the World Health Organization Classification of Lymphoid Neoplasms [34].

In order to compare our results with available data at a global level, we interrogated published information on CMV prevalences and burden of B-cell malignancies across the globe. The PubMed advanced search was used with the search keywords "cytomegalovirus", "CMV", "B lymphoma", "Hodgkin’s disease", "non-Hodgkin lymphoma", "B acute lymphoblastic leukemia", "B chronic lymphocytic leukemia" and "myeloma", all being neoplasms of the B-cell lineage.

Incidences of B-cell malignancies were obtained from the World Health Organization Global Cancer Observatory (GLOBOCAN) [35] and compared to CMV prevalences from 74 countries for which this data was available [36]. Age-adjusted annual incidence rates (/10^5^ population) of B-cell neoplasms (standardized to the year 2000 US Census Bureau million population by the direct method) were collected and presented as sums rather than separately and apart.

It is important to note that published reports do not always clearly discriminate between B-cell and T-cell disorders. Cases of B- and T-acute lymphoblastic leukemia (B-ALL and T-ALL) were frequently presented jointly as “ALL”. B- and T-non-Hodgkin's lymphoma (B-NHL and T-NHL) were often jointly described as “NHL”. Moreover, even if B- and T-cell components of lymphoproliferative diseases were reported, complementary information on the prevalence of CMV seropositivity for each component was often not reported. Crude annual incidence rates (/10^5^) of all B-cytopathies were summed-up in Tables 4 and 5. Merging the rates enhanced their statistical power and ease of interpretation.

**Table 4:** Provisional data on CMV seropositivity in summarized incidence rates of B-lymphoid neoplasms around the world

Population*	Representative CMV (%) seropositivity	B-neoplasms (merged rates yr^−1^/10^5^)**	*p*-value^†^	Refs
The US	44	44.72	N/A	Chihara et al*.* [55] SEER Program [56]
US-born Asians	75	18.6	< 0.05	Clarke et al*.* [52] Li et al*.* [53]
Europe	70	25.44	< 0.05	Zuhair et al*.* [36]
Middle East^¶^ (Iraq, Jordan, S. Arabia)	90	9.12	< 0.05	Yaqo et al*.* [57]
Sub-Saharan Africa	~ 92	7.2	< 0.05	Tomoka et al*.* [81]
Hong Kong	> 90	11.49	< 0.05	Bassig et al*.* [58]
East Asia (China, Japan)	~ 90	8.72	< 0.05	Chihara et al*.* [55]

*Males and females were combined. Worldwide population means of CMV seroprevalence by the International Agency for Research on Cancer (IARC) [35] were relied on in comparisons with annual rates of B-cell maladies. We made use of standardized population-based cancer registry records in Chihara et al.[55]

**The crude numbers of extracted incidence rates of B-cell neoplasms were merged together in the middle column). Compounded incidences were preferred as a proviso for each world zone. Thus, rough rates depart from factual ones and should be taken as broadly consultative. Also, racial groups were collected under different protocols, excepting the Surveillance, Epidemiology, and End Results (SEER) 13 Incidence Database. Withal, unequal pathological categorization according to diagnosis (or cellular origin) and sub-classification criteria for B-cell leukemia/lymphoma in particular, varied from country to country introducing further biases. This hampered estimation of summated incidence rates. Consequently, workout of regional rates of B-cell neoplasms do not sum-up to estimates reported in the literature used

^†^*P-values* were two-sided using the *t*-test. They reflect coupling strength between CMV seropositivity and incidence rate of B-cell neoplasms in the US versus other countries

^¶^Age-adjusted incidence rates refer to patients > 70 years of age. This age group had the longest exposure to CMV and could not be strictly compared to global SEER data

Remark: The differences in study populations, high false discovery rates and dissimilar calendar periods of observation in the reports used affected the calculations. These should be viewed with caution

**Table 5:** Crude incidence rate estimates of key B-cell malignancies: aggregate rates by country, race, and ethnicity

Country (race/ethnicity)	Lymphoid malignancy (crude annual incidence estimates/10^5^ population)					Summed crude aggregate estimates
	B-NHL*	HD	B-ALL	B-CLL	PCM	
The US	27.4	3.23	1.69	6.3	6.1	44.72
The US-born Asians	11.8	1.28	0.7	1.8	3.2	18.6
Europe (wgt. mean)	11.6	3.58	0.68	4.88	4.7	25.44
Middle East	4.9	1.45	0.56	0.71	1.5	9.12
Sub-Saharan Africa	5.5	0.7	0.35	0.36	0.29	7.2
Hong Kong	6.68	0.62	1.28	0.6	2.31	11.49
East Asia (China, Japan)	4.8	0.53	0.96	1.13	1.3	8.72

*B-NHL B-cell non-Hodgkin Lymphoma; *HD* Hodgkin’s disease; *B-ALL* B-cell acute lymphoblastic leukemia; *B-CLL* B-cell chronic lymphocytic leukemia; *PCM* plasma cell myeloma

Clinical sampling was approved by University Clinical Centre of Serbia, University of Belgrade Ethical Review Board. The patients signed individually a document of informed consent.

### Statistical analysis

Results are presented as count (percent) or median (min–max) depending on data type. Groups were compared using non-parametric tests, Fisher's exact test for frequencies, Mantel–Haenszel chi square test for trend and Mann–Whitney U test for numeric data with non-normal distribution. Propensity score matching was performed in order to find the best matching cases in control group by age and gender. Correlation between numerical variables was performed using Spearman correlation analysis. All *p-*values less than 0.05 were considered significant. All data were analyzed using SPSS 20.0 (IBM corp.) statistical software.

## Results

General characteristics of the patient group are presented in Tables 1 and 2 and the comparisons between the study and control groups is presented in Table 3. Tests for IgG antibodies were successful in all patients.

CMV serostatus was relatively homogeneous across different B-cell neoplasms despite their glaring clinical diversity which ranged from acute B-ALL and aggressive B-NHL to mature B-chronic lymphocytic leukemia (B-CLL), low-grade B-NHL, and plasmocytoma (Table 1). Biological characteristics they share in common (immunophenotype and somatic mutation profiles) remain preserved in cancerogenesis such that clinical distinctiveness of B-cell neoplasms did not hamper the understanding of their virology.

Most IgG positives were patients with NHL (33/35, 94.3%) followed by B-CLL (8/9, 88.9%), and Hodgkin's disease (HD) (13/17, 76.5%). CMV was least pervasive in multiple myeloma (2/3, 66.7%) but the patients were too few. Low natural incidence of some B-cell disorders resulted in a low number of consecutive patients detected over a short interval of observation. All patients with hairy cell leukemia (2/2), Waldenström's macroglobulinemia (2/2), and non-specified B-cell lymphoma (2/2) were IgG positive. Their numbers were insufficient and were excluded from separate analyses. Positive CMV serology did not correlate among different B-lymphoproliferative diseases (*p* = 0.339).

The study cohorts had markedly different (*p* < 0.001) age and gender structure (Table 3). This required statistical matching to compensate for these discrepancies, after which there remained statistical variance for neither of variables (Table 3). Interestingly, a notable difference in CMV seropositivity emerged between the study group and normal populace after the gender/age matching was performed. The prevalence of CMV infection was significantly higher in the control group (*p* = 0.035), compared to the patient group (Table 3).

Binary logistic regression with B-cell malignancy as dependent and CMV serostatus as independent variable demonstrated that subjects with positive serostatus were ~ 7 times less likely (OR, 0.067; 95% CI, 0.016 to 1.150) to have a B-cell malignancy relative to seronegatives. The difference was not significant (*p* = 0.067), but near the conventional level of significance (0.05).

**Fig. 1:**
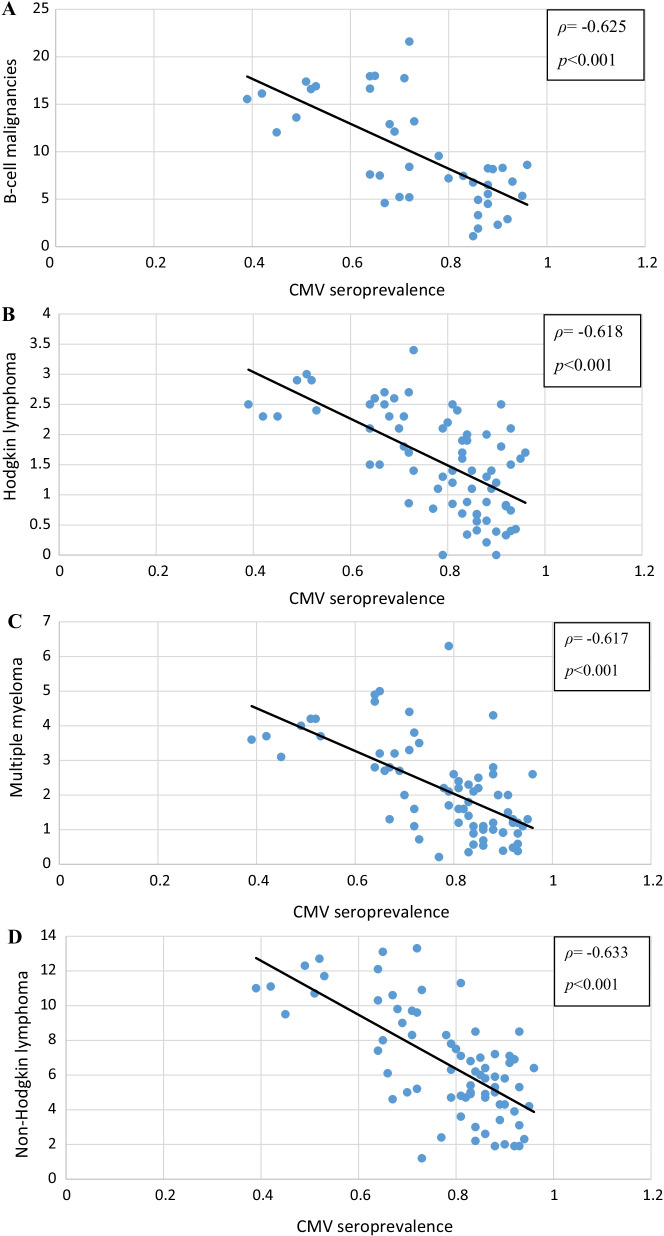
The scatter charts present country specific CMV prevalence (mean) plotted against estimated age-standardized (world) annual incidence rates (per 100,000) of microscopically verified cases of B-cell types of cancer in 74 countries (blue circles) [35, 36, 69]. **A**) B-cell malignancies (all types) (Spearman *ρ* = -0.625, *p* < 0.001), **B**) Hodgkin’s disease (Spearman *ρ* = -0.618, *p* < 0.001), **C**) non-Hodgkin lymphomas (Spearman *ρ* =  = -0.617, *p* < 0.001), and **D**) multiple myeloma (Spearman *ρ* = -0.633, *p* < 0.001) in 2020. The inverse relationship between viral pervasiveness and the annual incidence rate of hematologic malignancies is highly significant for all (**A**) and each individual B-cell cancer type (**C**-**D**)

The results pointed to a potential protective effect that CMV may proffer against B-cell dyscrasia. In order to investigate our evidence on a much larger scale, we compared annual incidence rates of B-cell neoplasms to CMV prevalences in 74 countries for which these variables were available (Fig. 1A–D). Interestingly, a significant negative correlation between CMV pervasiveness and the incidence of all clinical types of B-cell malignancies was observed the world over (Fig. 1A; Spearman *ρ* = −0.625, *p* < 0.001). Similarly, an inverse association was evidenced separately for three different B-cell malignancies: HD (Fig. 1B; Spearman *ρ* = −0.618, *p* < 0.001), non-Hodgkin lymphomas (Fig. 1C; Spearman *ρ* = −0.617, *p* < 0.001), and myeloma (Fig. 1D; Spearman *ρ* = −0.633, *p* < 0.001), separately.

These results support the reality of oncoprotection by the chronic CMV infection against B-lymphomagenesis irrespective of a clinical form of a B-cell neoplasm.

## Discussion

This is the first study reporting on the current estimate of CMV infection in Serbian hemato-oncological patients and healthy controls. Also, our clinical results are supported by the worldwide survey of relevant data. Together, they offer the first insight into a possible connection between the chronic CMV infection and B-cell neoplasms, hinting at an oncoprotection conferred by this virus on its host.

### CMV seroprevalences in patients with hematological malignancies

CMV seroprevalence varies in published studies on patients with hematological malignancies. Virus prevalence in our patient cohort (90.4%) places the Republic of Serbia among the most CMV-permeated populations in the world [37–44]. Much lower seroprevalence (70%) of anti-CMV IgG was reported in a multicenter cohort of Swedish patients (Re: Mission, NCT01347996, www.clinicaltrials.gov [45]. The *lowest* CMV infestation was reported in landmark studies from the US [46, 47], a highly developed country with one of the largest incidence rates of B-cell disorders.

In studies on HCT recipients [3], and B-CLL patients [48], females were significantly more CMV seropositive. Similar to our clinical population, in Brazilian patients with various hematologic disorders females were more CMV seropositive than males albeit not significantly [37]. On the contrary, Sudan females with leukemia were less seropositive for CMV than males [49]. Marchesi et al*.* [39] reported largest prevalence of CMV in patients with B-CLL, and multiple myeloma which is similar to the present findings.

### Inverse association between CMV seroprevalence and incidence of B-cell neoplasms across the globe

There is a stark difference in annual incidences of B-lymphoid malignancies between Western and Eastern countries [50, 51]. We try to draw attention to an inverse association between the annual age-adjusted incidences of B-cell malignancies and the spread of CMV seropositivity at a global level (Tables 5 and 6, Fig. 1A–D). Seroprevalence in presumably epidemiologically unrelated communities was frequently lower in patients with B-cell and even in other malignancies (acute myeloid leukemia, AML; chronic myeloid leukemia—CML) than that reported in voluntary blood/organ donors and in the general population [36]. This difference is explainable if chronic CMV infection conferred a degree of protection on its immunocompetent host against B-cell malignancies. This is consistent with the evidence in the current work where healthy controls were significantly more CMV seropositive (*p* = 0.035; Table 3) than patients with B-cell malignancies. A potential explanation might be an increase in resistence against B-cell neoplasia fostered by primary CMV infection.

**Table 6:** Country-specific CMV seroprevalence in patient cohorts compared to matched blood/organ donors and healthy general populations

Continent, country, or region (listed West-to-East)	Hematologic malignancy	Cytomegalovirus seropositivity (CMV^+^)			Transplant type and support medication	References
		Patients N, (CMV^+^ %) (unless otherwise noted)	Country specific blood/organ donors CMV^+^, mean [36]	Country specific general popul. CMV^+^, mean [36]		
*North & South America*						
USA, Boston, New York Washington	ALL	**1 359 (48%)**	**0.67**	**0.64**	Allo-HCT, GvHD prophylaxis	Kollman et al*.* 2001 [46]
USA, Seattle	AL, NHL, HD, MM, CLL	**835 (49%)**	**0.67**	**0.64**	Allo-HCT, GvHD prophylaxis	Nichols et al*.* 2002 [47]
USA, Arkansas	MM, HD	**107 (57%)**	**0.67**	**0.64**	Auto-HCT, BCNU, BEAM, melphalan alone	Fassas et al*.* 2001 [82]
USA, Texas	ALL, CLL, NHL	**680 (52.4%)**	**0.67**	**0.64**	Chemo radiation therapy	Nguyen et al*.* 2001 [83]
USA, The Netherlands, transplant centers	ALL	**952 (47.5%)**	**0.63**	**0.60**	Allo-HCT, GvHD prophylaxis	Lee et al*.* 2007 [84]
USA, California	AL (children)	**144 (55.7%)**	**0.67**	**0.64**	Allo-HCT, GvHD prophylaxis	Behrendt et al*.* 2009 [85]
Brazil, Bahia State	Hematology patients (various)	**470 (89.4%)**	**0.91**	**0.89**	N/A	De Matos et al*.* 2011 [37]
Brazil, Campinas, SP	ALL, MM, HD, NHL,	**20 (82.5%)**	**0.91**	**0.89**	Allo-HCT, GvHD prophylaxis	Dieamant et al*.* 2011 [86]
Chile, Santiago	Hematolgic malignancies (various)	**N/A (86%)**	**0.91**	**0.89**	HCT, GvHD prophylaxis	Ferrés et al*.* 2012 [87]
*America-Europe*						
Canada, US, UK, France (33 countries)	ALL (B- & T- cell types)	564 (59.7%)	0.55 (ave.)	0.51		Duval et al*.* 2010 [88]
Canada, US, UK, Saudi Arabia, The Netherlands (CIBMTR), ~ 200 transpl. centers	ALL (B- & T- cell types)	1 883 (59.7%)	0.62 (ave.)	0.59 (ave.)	Allo-HCT GvHD prophylaxis	Teira et al*.* 2016 [89]
46 international transpl. centers	Poor risk ALL (B- & T-cell types)	**127 (47%)**	**0.63**	**0.58**	MUD-BMT	Cornelissen et al*.* 2001 [90]
67 international transpl. centers (20 countries)	ALL, NHL, MM, CLL	**165 (60% donors)**	**0.66 (ave.)**	**0.68 (ave.)**	Allo-HCT	Marty et al*.* 2017 [91]
*Europe*						
Ireland, Dublin	Hematologic malignancies (various)	72 (48%)	0.43	0.39	Allo-HCT (43 pts.) Chemotherapy (28 pts.)	Fleming et al*.* 2010 [92]
Sweden & Italy, Gothenburg, Rome	AML	**81 (70%)**	**0.74**	**0.71**	Chemoradio therapy	Bernson et al*.* [45]
Sweden, Germany, The Netherlands, Italy, France	ALL (B- &T- cell types)	**3 539 (55%)**	**0.62 (ave.)**	**0.58 (ave.)**	Allo-BMT & HCT	Ljungman et al*.* 2003 [2]
EBMT member centers (whole registry cohort)	ALL, AML, HD, NHL	**40 306 (53.9%)**	**0.62 (ave.)**	**0.58 (ave.)**	Allo- & auto-HCT	Ljungman & Brand 2007 [3]
Sweden, Spain, UK, France, Italy, The Netherlands, Poland, Germany	ALL, LY	**31 669 (59.8%)**	**0.62 (ave.)**	**0.58 (ave.)**	Allo-HCT	Ljungman et al*.* 2014 [93]
48 transpl. centers (Europe, MENA, South Africa)	ALL, NHL, HD	165 (81.4%)	0.77 (ave.)	0.75 (ave.)	Allo-BMT	Ljungman et al*.* 2002 [94]
US (CIBMTR), Canada, UK, Spain, Sweden, Saudi Arabia, New Zealand, Japan	Childhood ALL (B- & T-cell)	**980 (53%)**	**0.69**	**0.65**	MRD, URD, UCBT, PB-HCT from URD	Mehta et al*.* 2019 [95]
France, USA, UK, Germany, Czech Republic, Israel	AML (age > 50)	3 398 (65%)	0.60 (ave.)		MUD HCT	Rubio et al*.* 2016 [96]
11 European countries with Israel and Turkey	Childhood B-precursor ALL	**140 (41%)**	**0.63 (ave.)**	**0.65 (ave.)**	Allo-HCT	Dalle et al*.* 2018 [97]
France, Israel, Spain, Germany, Belgium, The Netherlands, UK, Poland (EBMT)	B-ALL (age > 60)	126 (59.3%)	0.61	0.57	RIC allo-HCT	Roth-Guepin et al*.* 2017 [98]
Belgium, Brussels	n.m (9 children, 7 adults)	**16 (35%)**	**0.56**	**0.52**	Allo-HCT	Debaugnies et al*.* [99]
Denmark, Copenhagen	Childhood ALL & adults	**118 (54%)**	**0.60**	N/A	Allo-HCT	Kielsen et al*.* 2018 [100]
The Netherlands, Utrecht	ALL, NHL, HD	**101 (50.6%)**	**0.57**	**0.53**	Allo-BMT	Meijer et al*.* 2002 [101]
The Netherlands, Rotterdam	ALL, NHL, MM	47 (66%)	0.57	0.53	Allo-HCT (sibling)	Broers et al*.* 2000 [102]
Poland, Wroclaw	ALL, LY	**26 (78%)**	**0.7**	**0.66**	HCT	Jaskula et al*.* 2015 [103]
Czech Republic, Brno	Childhood & adolescent ALL, NHL, HD	**104 (37.6%)**	**n.m**	**0.42** (healthy age-matched control)	Conventional chemotherapy	Michálek & Horvath 2002 [104]
Germany, Russian Federation, Hamburg, St. Petersburg	ALL, NHL	**54 (39%)**	**0.62**	**0.57**	Allo-HCT	Kröger et al*.* 2001 [105]
Russian Federation, Moscow	AML, Mantle cell lymphoma	**183 (45.9%)**	**0.74**	**0.7**	Allo-HCT	Vdovin et al*.* 2016 [106]
Croatia, Zagreb	ALL, NHL, MM, HD, CLL	**47 (77%)**	**0.83**	**0.8**	Allo-HCT	Peric et al*.* 2018 [107]
Hungary, Szeged	LY	**224 (75%)**	**0.87**	**0.84**	Auto-HCT Chemotherapy	Piukovics et al. 2017 [1]
Italy, Rome	AML	52 (93%)	0.76	0.73	Chemoradio therapy	Capria et al*.* 2010 [40]
Italy, Rome	LY	327 (93%)	0.76	0.73	Auto-BMT & Auto-HCT	Marchesi et al*.* 2015 [39]
Italy, Milan, Udine, Bergamo, Ancona, Alessandria	B-cell lymphoma	**265 (70%)**	**0.76**	**0.73**	Allo-HCT	Mariotti et al*.* 2014 [108]
Germany, France, Finland	ALL (B- & T-cell)	5 158 (60%)	0.58	0.54	Allo-HCT	Schmidt-Hieber et al*.* 2013 [109]
Serbia, Belgrade-Srem-Šumadija	ALL, HD, WD, CLL, MM,	**83 (88%)**	**N/A**	**0.9**	Allo-HCT, Chemoradio therapy	this work
Spain, Barcelona	AL, NHL, CLL, MM, HD/ST	**150 (66%)**	**0.72**	**0.69**	Allo-HCT-RIC GvHD prophylaxis	Piñana et al*.* 2010 [110]
*Middle East North Africa Region*						
Kingdom of Saudi Arabia, Jeddah	ALL, NHL, HD, MM, CLL	1 252 (95.76%)	0.89	0.88	Chemothrapy HCT	Zaidi et al*.* 2019 [41]
Kingdom of Saudi Arabia, Jordan, Riyadh, Irbid	ALL (children)	**82 (1.22%)**	**0.85**	**0.87**	Allo-HCT UCBT GvHD prophylaxis	Al-Sweedan et al*.* 2017 [111]
Kingdom of Saudi Arabia, Riyadh	AL (children)	**73 (68%)**	**0.89**	**0.88**	Allo-cord blood HCT	Al-Hajjar et al*.* 2011 [112]
Jordan, Amman	AL (children)	**72 (31%)**	**0.83**	**0.85**	Auto-HCT	Hussein et al*.* 2015 [113] & Al Mana et al*.* 2019 [114]
Israel, Tel Aviv, Petah Tikva	AL, LY	**121 (61%)**	**0.75**	**0.72**	Allo-HCT GvHD prophylaxis	Cohen et al*.* 2015 [115]
Iran, Tehran, Rasht	ALL, AML, NHL, MM & various disease	126 (97.6%)	0.96	0.95	Allo-HCT GvHD prophylaxis	Valadkhani et al*.* 2016 [116]
Iran, Mashhad	Voluntary blood donors	1 000 (99.2%)	0.96	0.95	N/A	Safabakhsh et al*.* 2013 [117]
Iran, Urmia	End-stage renal disease (immunodeficiency)	**65 (77.4%)**	**0.96**	**0.95**	Pre-transplant hemodialysis	Sepehrvand et al*.* 2010 [118]
Iran, Shiraz, Tehran,	Leukemia (unspecified)	**6 (100%)**	**0.96**	**0.95**	Allo-BMT	Behzad-Behbahani et al*.* 2004 [119]
Iran, Tehran, Shiraz	AML, Thalassemia, CML, AA, ALL	26 (100%)	0.96	0.95	BMT	Ziyaeyan *et. al.* 2006 [120]
Tunisia, Sousse, Sfax	ALL	**39 (90%)**	**0.94**	**0.93**	Chemotherapy (different phases)	Handous et al*.* 2020 [121]
Egypt, Alexandria	Voluntary blood donors	88 (96.6%)	0.94	0.93	N/A	Gawad et al*.* 2016 [122]
Egypt, Cairo	AML & ALL	28 (39%, active CMV)	0.94	0.93	BMT	Zekri et al*.* 2004 [123]
Egypt, Cairo	B-ALL (40) T-ALL (10) (children & adolesc.)	**40 (36% CMV DNA/serum)**	**30 (46.7% CMV DNA control)**	**0.93**	Consolidation Tx Salvage Tx	Loutfy et al*.* 2017 [124]
*Australia, India*						
Australia, Victoria	AL, B-NHL, HD, CLL, MM	28 (88%)	0.69	0.65	Conventional-dose chemotherapy Auto-HCT	Ng et al*.* 2005 [38]
Australia, Sydney	ALL, NHL, MM	**103 (63%)**	**0.69**	**0.65**	Allo-HCT, MUD-HCT	George et al*.* 2010 [125]
India, Vellore	Malignant & non-malignant diseases Patients: Donors:	463 (97.4) **403 (84.8%)**	**0.88**	**0.86**	Allo-HCT	Devasia et al*.* 2018 [126]
*East Asia*						
Malaysia, Australia. Kuala Lumpur, Melbourne	ALL N = 71, AML N = 6, med. age: 28 yr	**77 (73%)**	**0.78 (ave.)**	**0.75 (ave.)**	Chemoradiotherapy	Azanan et al*.* 2016 [127]
China, Guangzhou province	B-ALL, B-NHL	**156 (86%)**	**0.92**	**0.9**	Allo-HCT (intensified condit.)	Xuan et al*.* 2012 [42]
China, Beijing	ALL, CML, MDS, AA, NHL	**60 (87%)**	**0.92**	**0.9**	Allo-HCT	Du et al*.* 2007 [128]
Taiwan, Kaohsiung, Taipei	AL, NHL	**117 (91.8%)**	**0.95**	**0.93**	Allo-HCT	Liu et al*.* 2012 [43]
Japan, Tokyo	Childhood AL	184 (81%)	0.76	0.72	UCBT	Tomonari et al*.* 2008 [44]
Japan, Fukuoka	Childhood ALL	**101 (72%)**	**0.76**	**0.72**	Allo-HCT	Inagaki et al*.* 2015 [26]

*n.m.* not mentioned; *N/A* not applicable; *AL* denotes acute leukemia (*AML* and *B-* and *T*-cell *ALL* together); *AML*, acute myeloblastic leukemia; *ALL*, acute lymphoblastic leukemia; *NHL*, non-Hodgkin lymphoma (B- and T-cell); *HD*, Hodgkin's disease; *MM*, multiple (plasma cell) myeloma; *CLL*, chronic lymphocytic leukemia; *LY*, lymphoid neoplasms (*HD*, *NHL* of *B-* and *T-*cell types); *WD*, Waldenström's disease; myeloid neoplasms (*AML*; *CML*, chronic myeloid leukemia; *MDS*, myelodysplastic syndrome; *MPN*, myeloproliferative neoplasm); *AA*, aplastic anemia; *allo-HCT*, allogeneic hematopoietic cell transplant; *auto-HCT*, autologous *HCT*; *MRD*, matched related donor; *URD*, unrelated donor; *UCBT*, umbilical cord blood transplantation; *PB-HCT*, peripheral blood *HCT*; *GvHD*, graft *vs.* host disease; *BMT*, bone marrow transplantation; *MUD*, matched unrelated donor; *BCNU*, *BEAM*, and melphalan indicate myeloablative protocols; *CIBMTR*, Center for International Bone Marrow Transplant Research; *MENA*, Middle East North Africa; *RIC*, reduced intensity chemotherapy *HCT*; *ST*, solid tumors

Studies with significantly lower CMV seropositivities in auto- and allo-HCTed patients, as compared to healthy donors and country-specific CMV prevalences, have seropositivity values indicated in bold. This was to point out the studies evidencing a reduced CMV protection against lymphoproliferation in the patients reported. CMV seroprevalence in patients with hematological diseases across racial and ethnic groups divisions is presented West-to-East. In most studies with mixed disease settings the prevalence of CMV seropositivity in subsets of B-cell diseases was not available. This perturbed the estimates of CMV seropositivity of interest, affecting the veracity of presented data. Notwithstanding partially inadequate representation of patient populations, the prevalence of CMV in hematologic malignancies shown remains mostly lesser than the corresponding country means [36]. This may signal a certain degree of oncoprotection secured to chronic carriers of latent virus. Endemicity of CMV seems to depend on SES defined factors and correlates with the incidence rate of malignant B-cell diseases across distant domains (Tables 4 and 5, Fig. 1 A‒D)

As the prevalence of CMV infection recedes across the populations, corresponding annual incidence of B-cell diseases tends to increase. For decades, incidence of lymphoid neoplasms has been globally increasing across age strata and sex. This may signify a gradual loss of protection provided by the latent CMV infection which is being globally eroded by steadily improving economic prowess and modern access to health care.

A racial/ethnic background is related to SES [52, 53]. The difference in incidence of B-lymphoid malignancies between the US and Japan is elevated, 2.5- to fivefold. The largest proportional difference between the US and Japan was in B-CLL (the US, 24.1%; Japan, 3.2%) [52]. Annual incidence rates of B-cell neoplasms in the US-born Asians/Pacific islanders are generally intermediate to those in the US whites and East Asians; exactly parallel trend is observed in their respective CMV seroprevalences. The incidence rates of B-cell neoplasms tend to negatively parallel the prevalence of CMV seropositivity in respective populations worldwide (Fig. 1A‒D). HD and B-NHL showed the largest difference in annual incidences between the US and East Asian countries. The SES correlates with trends in age-standardized incidences of B-lymphoid disorders and is also associated with CMV infection around the world. Seroprevalence of CMV decreased in pregnant women in Ishikawa Prefecture (Japan) from 93.2% to 66.7% over the period between 1980 and 1998 and in parallel with the increase in SES [54]. Of note, age-adjusted incidence of lymphoid malignancies in Japan increased significantly as opposed to no significant annual percent change in the US (Japan, + 2.4%; US, + 0.1%) [55]. This may be a consequence of growing SES in Japan and the consequent drop in CMV infection there.

Global disease burden reports [36, 52, 53, 55–72] suggest a significant inverse correlation between overall estimates of CMV seropositivity and the age-standardized and population-based incidence rate of B-cell cancers (Tables 4 and 6, Fig. 1A–D). Cytomegalovirus infection decreases as contemporary economy improves and affluence is gained across societal strata. Reduced rates of CMV primoinfection in developed countries may be the cause of an increased risk of contracting a B-cell malignancy. By contrast, high CMV prevalence in countries with adverse economic conditions, appears to mitigate the risk of B-lymphoproliferative disease. In populations where the prevalence of CMV declines an oncoprotective effect of CMV subsides such that an increased annual incidence rate of B-cell cancer is observed worldwide (Spearman *ρ* = -0.625, *p* < 0.001). However, some other factor(s) may operate along with CMV infection influencing the global correlation between increasing incidence of B-cell malignancies, improving SES, and reduced country-specific prevalence of CMV infection.

### *Clinical and *in vitro* experimental evidence supporting oncoprotection by CMV*

Cytomegalovirus seroprevalence was higher in the controls than in our patients with B-cell malignancies (Table 3; *p* = 0.035). This argues against the promotive contribution of CMV in B-cell lymphomagenesis.

Evidence in favor of viral repression of the transformation process in cancer cells has been reported [73]. CMV inhibits the migratory capacity of mesenchymal breast cancer cell lines MDA-MB-231 and SUM1315 [19]. Mice xenografted with CMV-infected HepG2 cells were reported to manifest limited to no tumor growth, as opposed to an unbridled tumor expansion in placebo-treated mice [74]. A runaway tumor growth was inhibited by restricting STAT3 activation, as well as by activation of the intrinsic apoptotic pathway [74, 75]. Apoptosis was also registered in the lung tissue of xeno-engrafted mice where HepG2 cells infected with human CMV were administered subcutaneously [74]. Erlach et al*.* [21, 22] proposed an innate anti-tumor mechanism elicited by murine CMV infection involving apoptosis of a liver-adapted clonal variant of B-cell lymphoma. The murine CMV infection had a highly suppressive effect on lymphoma cells even without infecting them, resulting in a significant survival benefit. Erkes et al*.* [76] also demonstrated clearance of tumors in a mouse melanoma model after CMV was inoculated into growing neoplasm. Also, an inhibiting effect of CMV glycoprotein B on breast cancer cell migration was recently documented by Yang et al.[20].

Anti-tumor effects of CMV infection were tentatively supported by reports of reduced relapse rates in patients with CMV reactivation early after allogeneic HCT for acute leukemia and NHL [23–27]. Changes within the immune system caused by CMV suggest a possible virus-*vs*-leukemia phenomenon [18] analogous to graft-*vs*-leukemia effect in B-CLL [77].

A study which screened neonatal Guthrie blood spots for CMV did not find that the CMV positives contracted B-ALL more often later in life [78]. MacKenzie et al*.* have screened common ALL patients and controls for presence of various herpesviruses, but were in doubt that a herpesvirus is an etiological agent in B-ALL [79]. Another study analyzed herpesvirus DNA in Guthrie cards and found no trace of EBV or HHV-6 but CMV presence has not been assessed [80]. Evidence garnered from these studies substantiates the assumption that CMV may forestall initiation of B-cell neoplasms.

A major strength of the present exploration is the use of a nationally representative sample to estimate CMV seroprevalence in the Republic of Serbia. Noteworthy limitations of our work are its retrospective nature and an artefact from a small sample size. Furthermore, a passive take of donor's IgG antibodies cannot be entirely excluded. This drawback to the study was mitigated by lower CMV seropositivity among blood transfusion-treated patients as compared to healthy controls.

## Conclusions

Conclusively, we present first set of data on CMV seroprevalence based on a sample of B-cell derived malignancies in Serbia. Also, we provide evidence that prevalences of CMV are strongly inversely associated with the annual incidence rates of malignant B-cell disorders the world over. This is suggestive of a possible protective effect of CMV against the profligate B-cell growth. The cellular niche may be less favourable for initiation of B-lymphomagenesis in chronic carriers of CMV. Prospective work with a larger study size of cell lineage-specific patient cohorts across clinical and histological lymphoma subtypes may be helpful in clarifying dilemmas regarding anti/pro tumoral activity of CMV.

## Data Availability

The datasets generated and/or analysed during the current study are not publicly available due to safeguarding patient anonymity, but are available from the corresponding author on reasonable request.

## Abbreviations

AA: Aplastic anemia
AL: Acute leukemia
ALL: Acute lymphoblastic leukemia
Allo-HCT: Allogeneic hematopoietic cell transplantation
AML: Acute myeloid leukemia
Auto-HCT: Autologous hematopoietic cell transplantation
BMT: Bone marrow transplant
CIBMTR: Center for International Bone Marrow Transplant Research
CLL: Chronic lymphocytic leukemia
CML: Chronic myeloid leukemia
CMV: Cytomegalovirus
GLOBOCAN: Global Cancer Observatory
GVHD: Graft-vs-host disease
HCT: Hematopoietic cell transplantation
HD: Hodgkin's disease
IARC: International Agency for Research on Cancer
LY: Lymphoid neoplasms
MDS: Myelodysplastic syndrome
MENA: Middle East North Africa
MM: Multiple myeloma
MPN: Myeloproliferative neoplasm
MRD: Matched related donor
MUD: Matched unrelated donor
n.m.: Not mentioned
N/A: Not applicable
NHL: Non-Hodgkin lymphoma
PB-HCT: Peripheral blood HCT
PCM: Plasma cell myeloma
PTLD: Posttransplant lymphoproliferative disorder
RIC: Reduced intensity chemotherapy
SEER: Surveillance, Epidemiology, and End Results
SES: Socioeconomic status
ST: Solid tumors
UCBT: Umbilical cord blood transplantation
URD: Unrelated donor
WD: Waldenström's disease
